# Cancer Stem Cells in Moderately Differentiated Buccal Mucosal Squamous Cell Carcinoma Express Components of the Renin–Angiotensin System

**DOI:** 10.3389/fsurg.2016.00052

**Published:** 2016-09-27

**Authors:** Therese Featherston, Helen H. Yu, Jonathan C. Dunne, Alice M. Chibnall, Helen D. Brasch, Paul F. Davis, Swee T. Tan, Tinte Itinteang

**Affiliations:** ^1^Gillies McIndoe Research Institute, Wellington, New Zealand; ^2^Wellington Regional Plastic, Maxillofacial and Burns Unit, Wellington, New Zealand

**Keywords:** buccal mucosa, squamous cell carcinoma, cancer, renin–angiotensin system, cancer stem cells, head and neck, oral cavity

## Abstract

**Aim:**

We have recently identified and characterized cancer stem cell (CSC) subpopulations within moderately differentiated buccal mucosal squamous cell carcinoma (MDBMSCC). We hypothesized that these CSCs express components of the renin–angiotensin system (RAS).

**Methods:**

3,3′-Diaminobenzidine (DAB) immunohistochemical (IHC) staining was performed on formalin-fixed paraffin-embedded MDBMSCC samples to investigate the expression of the components of the RAS: (pro)renin receptor (PRR), angiotensin converting enzyme (ACE), angiotensin II receptor 1 (ATIIR1), and angiotensin II receptor 2 (ATIIR2). NanoString mRNA gene expression analysis and Western Blotting (WB) were performed on snap-frozen MDBMSCC samples to confirm gene expression and translation of these transcripts, respectively. Double immunofluorescent (IF) IHC staining of these components of the RAS with the embryonic stem cell markers OCT4 or SALL4 was performed to demonstrate their localization in relation to the CSC subpopulations within MDBMSCC.

**Results:**

DAB IHC staining demonstrated expression of PRR, ACE, ATIIR1, and ATIIR2 in MDBMSCC. IF IHC staining showed that PRR was expressed by the CSC subpopulations within the tumor nests, the peri-tumoral stroma, and the endothelium of the microvessels within the peri-tumoral stroma. ATIIR1 and ATIIR2 were localized to the CSC subpopulations within the tumor nests and the peri-tumoral stroma, while ACE was localized to the endothelium of the microvessels within the peri-tumoral stroma. WB and NanoString analyses confirmed protein expression and transcription activation of PRR, ACE, and ATIIR1, but not of ATIIR2, respectively.

**Conclusion:**

Our novel findings of the presence and localization of PRR, ACE, ATIIR1, and potentially ATIIR2 to the CSC subpopulations within MDBMSCC suggest CSC as a therapeutic target by modulation of the RAS.

## Introduction

Oral cavity cancer is the sixth most common cancer globally (1, 2) with more than 90% being squamous cell carcinoma (SCC) (2, 3). Oral cavity SCC (OCSCC) arises from the squamous epithelium of the lips, oral tongue, floor of mouth, hard palate, buccal mucosa, maxillary and mandibular alveolus, and the retromolar trigone (1, 4). Predisposing factors for BMSCC include tobacco use, alcohol abuse, and betel leaf chewing (5). BMSCC is most prevalent in South East Asia and Southern Asia due to the habitual use of betel quid and betel leaf chewing (2, 4), more commonly in men (2).

Current treatment for BMSCC involves surgery, often with postoperative radiotherapy (RT), and sometimes chemotherapy (ChT) (2). Despite advances in treatment, 5-year survival for BMSCC remains 50–58%, and the overall survival has only increased by 5% in the past 20 years (1, 4, 6). This poor prognosis is partly due to late presentation with advanced loco-regional disease and less commonly metastasis to the bone, brain, or liver (5).

Cancer stem cells (CSCs) have been demonstrated in many types of cancers and have been proposed to be the origin of these cancers, including BMSCC (7). CSCs are suggested to play a crucial role in carcinogenesis with their ability for self-renewal and differentiation into multiple lineages through symmetric and asymmetric division, giving rise to diverse cell populations (7).

Although the origin of CSCs remains unclear (8), they are distinguished from the majority of the cancer cell population by their expression of CSC markers (9). Overexpression of CSC markers has been associated with increased tumor size, local invasion, and metastasis (10, 11). Increased expression of CSC markers has also been associated with worse prognosis (12–14), treatment resistance, and higher risk of loco-regional recurrence and distant metastasis following RT and ChT (15).

We have recently demonstrated the presence, within moderately differentiated buccal mucosal squamous cell carcinoma (MDBMSCC), of an EMA^+^/SOX2^+^/SALL4^+^/OCT4^+^/pSTAT3^+^/NANOG^+^ CSC subpopulation within the tumor nests; as well as two separate peri-tumoral stromal populations one expressing EMA^−^/SOX2^+^/OCT4^+^/pSTAT3^+^/NANOG^+^ and another expressing EMA^−^/CD34^+^/SOX2^+^/OCT4^+^/pSTAT3^+^/NANOG^+^ localized to the endothelium of the microvessels (16). Intriguingly, this unique expression pattern parallels our recent findings in moderately differentiated oral tongue SCC (OTSCC) (17). The renin–angiotensin system (RAS) is a hormonal system that is classically associated with blood pressure regulation (18, 19). A key component of the RAS is angiotensinogen (ANG) that is physiologically released from the liver into the circulation (20). ANG is converted to angiotensin I (ATI) by renin – the active form of the proenzyme, pro-renin (18, 21). The receptor for both renin and its proenzyme is known as the (pro)renin receptor (PRR) (22). ATI is then converted to angiotensin II (ATII) by angiotensin converting enzyme (ACE) (18). The effects of the vasoactive ATII are mediated through its receptors, angiotensin II receptor 1 (ATIIR1) and angiotensin II receptor 2 (ATIIR2) (18).

We have previously demonstrated the role of stem cells in the biology of infantile hemangioma (IH) putatively regulated by the RAS (23, 24). This, coupled with recent publications, suggesting a role for the RAS in cancer growth (18), with components of the RAS: ACE, ATIIR1, and ATIIR2, being demonstrated in areas of cancer (25), led to the notion of RAS playing a role in tumor angiogenesis and tumor cell proliferation (25), both being determinants of tumor growth and metastasis. This suggests the RAS as a potential therapeutic target for cancer (18). Despite the proposed role of the RAS in carcinogenesis (18), there are currently no publications showing the presence of the RAS in BMSCC.

In this study, we investigated the expression of the components of the RAS: PRR, ACE, ATIIR1, and ATIIR2 within MDBMSCC using immunohistochemical (IHC) staining, Western Blotting (WB), and NanoString gene expression analysis. We also investigated the localization of these proteins in relation to the CSC subpopulations we have identified within this tumor (16).

## Materials and Methods

### Tissue Samples

Moderately differentiated buccal mucosal squamous cell carcinoma (MDBMSCC) specimens from one female and five male patients, aged 38–80 years (mean 59.3 years), sourced from the Gillies McIndoe Research Institute’s Tissue Bank, were used for this study, which was approved by the Central Regional Health and Disability Ethics Committee (ref. no. 12/CEN/74).

### Histochemical and Immunohistochemical Staining

Hematoxylin and eosin (H&E) staining was used to confirm the appropriate histological grade and to identify areas of MDBMSCC within the tissue sections by an anatomical pathologist (HDB). 3,3′-diaminobenzidine (DAB) IHC staining was performed on 4-μm thick formalin-fixed paraffin-embedded sections of six MDBMSCC samples using primary antibodies for SOX2 (1:500; cat# PA1-094, Thermo Fisher Scientific, Waltham, MA, USA), OCT4 (1:30; cat# MRQ-10, Cell Marque, Rocklin, CA, USA), epithelial membrane antigen (EMA, ready-to-use; cat# PA0212, Leica), CD34 (ready-to-use; cat# PA0212, Leica), PRR (1:2000; cat# ab40790, Abcam, Cambridge, MA, USA), ACE (1:100; cat# MCA2054, AbD Serotec, Raleigh, NC, USA), ATIIR1 (1:30; cat# ab9391, Abcam), and ATIIR2 (1:2000; cat# NBP1-77368, Novus Biologicals, Littleton, CO, USA). All slides were mounted in Surgipath Micromount (Leica).

To determine co-expression of the proteins, immunofluorescent (IF) IHC staining was performed on two samples of MDBMSCC from the original cohort of six patients used for DAB IHC staining, utilizing a combination of Vectafluor Excel anti-mouse 488 (ready-to-use; cat# VEDK2488, Vector Laboratories, Burlingame, CA, USA) and Alexa Fluor anti-rabbit 594 (1:500; cat# A21207, Life Technologies, Carlsbad, CA, USA) to detect combinations that included OCT4 and SALL4 with PRR, ACE, ATIIR1, and ATIIR2. All IF IHC-stained slides were mounted in Vecta Shield Hardset mounting medium with 4′,6′-diamidino-2-phenylindole (Vector Laboratories).

Positive human control tissues used for primary antibodies were placenta for PRR; liver for ACE, ATIIR1, and ATIIR2; and seminoma for OCT4 and SALL4. A negative MDBMSCC control sample was also prepared by omitting the primary antibodies.

All antibodies were diluted with Bond primary antibody diluent (cat# AR9352, Leica), and all DAB and IF IHC staining was carried out on the Leica Bond Rx autostainer as previously described (26).

### NanoString Gene Analysis

Five snap-frozen MDBMSCC samples from the original cohort of patients used for DAB IHC staining were utilized for isolation of total RNA for NanoString nCounter™ Gene Expression Assay (NanoString Technologies, Seattle, WA, USA). Extraction of RNA from tissues was performed using RNeasy Mini Kit (Qiagen, Hilden, Germany). RNA was quantitated by Qubit^®^ 2.0 Fluorometer (Life Technologies) and subjected to NanoString nCounter™ gene expression assay completed by New Zealand Genomics Ltd (Dunedin, NZ), according to the manufacturer’s protocol. Probes for the genes encoding for PRR (ATP6AP2, NM_005765.2), ACE (NM_000789.2), ATIIR1 (AGTR1, NM_000685.3), ATIIR2 (AGTR2, NM_000686.3), and the housekeeping gene GUSB (NM_000181.1) were designed and synthesized by NanoString Technologies. Raw data were analyzed by nSolver™ software (NanoString Technologies) using standard settings. Results were normalized against the housekeeping gene and graphed using Excel (Microsoft Office 2013).

### Western Blotting

Total protein was extracted from five MDBMSCC specimens by homogenization in ice-cold RIPA buffer (Sigma–Aldrich, St Louis, MA, USA) containing 10mM dithiothreitol (DTT) (Sigma–Aldrich) and 1× HALT protease and phosphatase inhibitor cocktail (Thermo Fisher Scientific*)*. Solubilized proteins were precipitated for 1 h at −20°C (ProteoExtract^®^ Protein Precipitation Kit, Merck Millipore, Billerica, MA, USA) and then re-suspended overnight in 1× Laemmli sample buffer (Bio-Rad, Hercules, CA, USA) containing 10mM DTT. Equal amounts of protein extracts were heated in sample buffer and then separated by Bolt™ 4–12% Bis-Tris Plus gel (cat# NW04120, Invitrogen, Carlsbad, CA, USA) electrophoresis. Separated proteins were then transferred to PVDF membrane (cat# IB24001, Life Technologies), which were then blocked in TBS containing 0.1% Tween-20 and 2% skim-milk powder for 90 min at 4°C. The membranes were subsequently probed using the following primary antibodies: PRR (1:500; cat# HPA003156, Sigma–Aldrich), ACE (1:200; cat# sc-12184, Santa Cruz Biotechnology, Dallas, TX, USA), ATIIR1 (1:200; cat# sc-1173, Santa Cruz Biotechnology), ATIIR2 (1:200; cat# ab92445, Abcam, Cambridge, MA, USA), and β-actin (1:1000; cat# ab82618, Abcam). Appropriate secondary antibodies used were goat anti-rabbit horseradish peroxidase (HRP) conjugate (1:2000; cat# A16110, Life Technologies), donkey anti-goat HRP conjugate (1:5000; cat# ab97120, Abcam), or fluorescent rabbit anti mouse Alexa Fluor^®^ 647 rabbit anti-mouse (1:2000; cat# A21239, Invitrogen). All primary and secondary antibodies were diluted in TBS containing 0.1% Tween-20 and 2% skim-milk powder. Detection of the HRP-conjugated secondary antibodies was achieved using Clarity™ Western ECL substrate (Bio-Rad). All membranes were imaged using the ChemiDoc MP imaging system (Bio-Rad).

### Image Analysis

3,3′-Diaminobenzidine IHC- and IF IHC-stained slides were viewed and imaged using an Olympus BX53 light microscope (Tokyo, Japan) and an Olympus FV1200 confocal laser-scanning microscope (Olympus), respectively. All IF IHC-stained images were processed with CellSens Dimension 1.11 software using the 2D deconvolution algorithm (Olympus).

## Results

### Histochemical and 3,3′-Diaminobenzidine Immunohistochemical Staining

**Figure 1:**
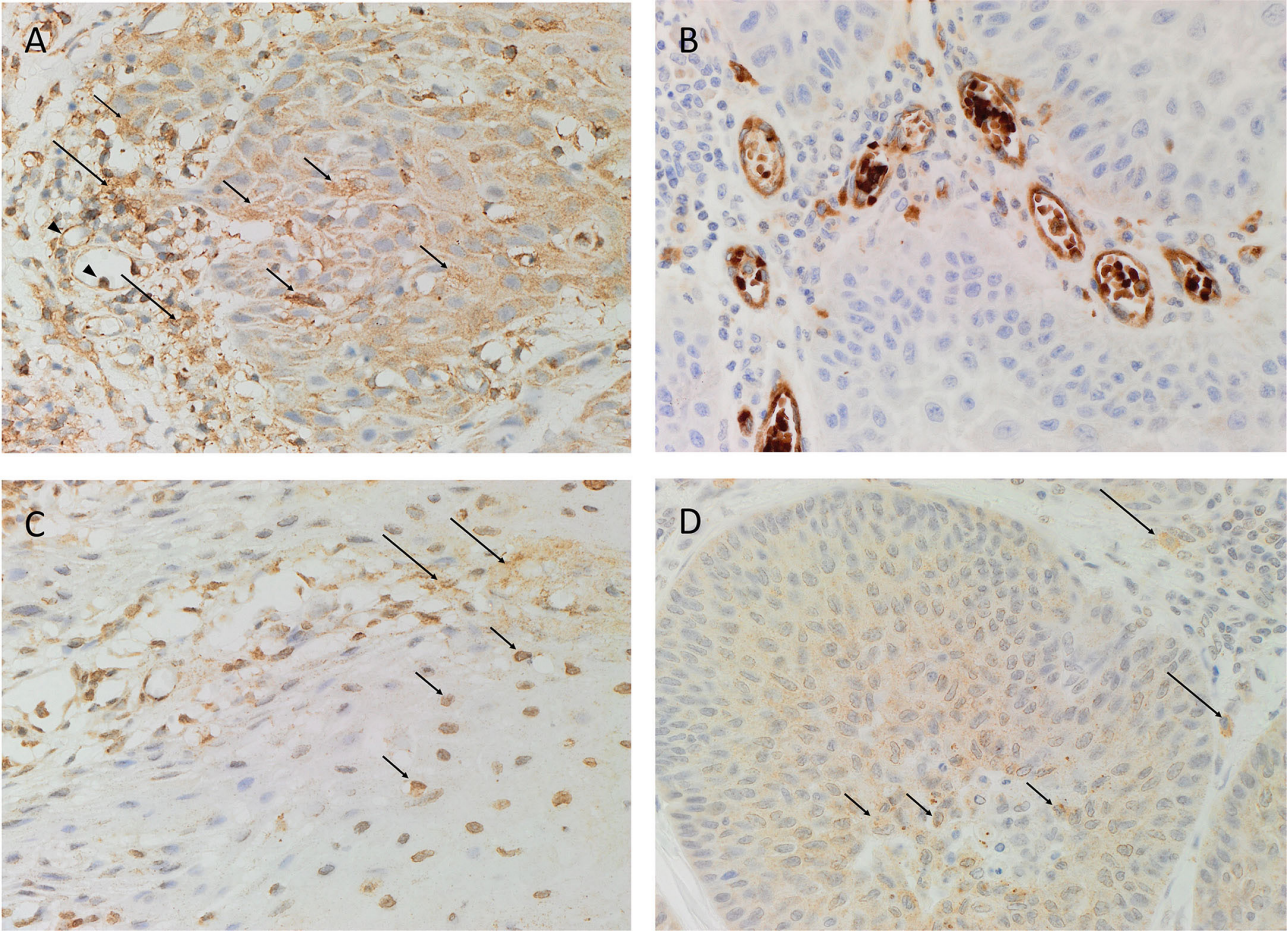
**Representative DAB IHC-stained sections of moderately differentiated buccal mucosal squamous cell carcinoma demonstrating cytoplasmic expression of PRR [(A), brown] by cells within the tumor nests (*short arrows*) and faint staining of endothelium (*arrowheads*) of the microvessels and peri-tumoral stroma (*long arrows*)**. ACE [**(B)**, brown] was expressed by the endothelium of the microvessels within the stroma. Strong cytoplasmic and nuclear expression of ATIIR1 [**(C)**, brown] was demonstrated in cells within the tumor nests (*short arrows*), with faint staining in cells within the peri-tumoral stroma (*long arrows*). Strong nuclear and faint cytoplasmic expression of ATIIR2 [**(D)**, brown] was demonstrated in cells within the tumor nests (*short arrows*) and some cells in the peri-tumoral stroma (*long arrows*). Nuclei were counterstained with hematoxylin [**(A–D)**, blue]. Original magnification: 400×.

Hematoxylin and eosin staining confirmed the histological grade and the presence of MDBMSCC (*n* = 6) for each sample. DAB IHC staining showed cytoplasmic expression of PRR (Figure 1A, brown) localized predominantly to cells within the tumor nests (Figure 1A, brown, *short arrows*). There was also faint immunoreactivity in the endothelium of the microvessels (Figure 1A, brown, *arrowheads*) and stronger cytoplasmic staining in cells within the peri-tumoral stroma (Figure 1A, brown, *long arrows*). ACE (Figure 1B, brown) was expressed on the endothelium of the microvessels, which were predominantly located around the periphery of the peri-tumoral stroma. ATIIR1 (Figure 1C, brown) was expressed predominantly in the cytoplasm and nuclei of cells within the tumor nests (Figure 1C, brown, *short arrows*) with faint cytoplasmic staining in cells within the peri-tumoral stroma (Figure 1C, brown, *long arrows*). Faint cytoplasmic and stronger nuclear staining of ATIIR2 (Figure 1D, brown) was observed in cells within the tumor nests (Figure 1D, brown, *short arrows*) and some cells within the peri-tumoral stroma (Figure 1D, brown, *long arrows*).

Positive controls for PRR, ACE, ATIIR1, and ATIIR2 demonstrated the expected staining patterns in human placenta (Image S1A in Supplementary Material, brown), liver (Image S1B in Supplementary Material, brown), liver (Image S1C in Supplementary Material, brown), and kidney (Image S1D in Supplementary Material, brown), respectively. The omission of the primary antibody in MDBMSCC samples provided an appropriate negative control (Image S1E in Supplementary Material, brown).

### Immunofluorescent Immunohistochemical Staining

**Figure 2:**
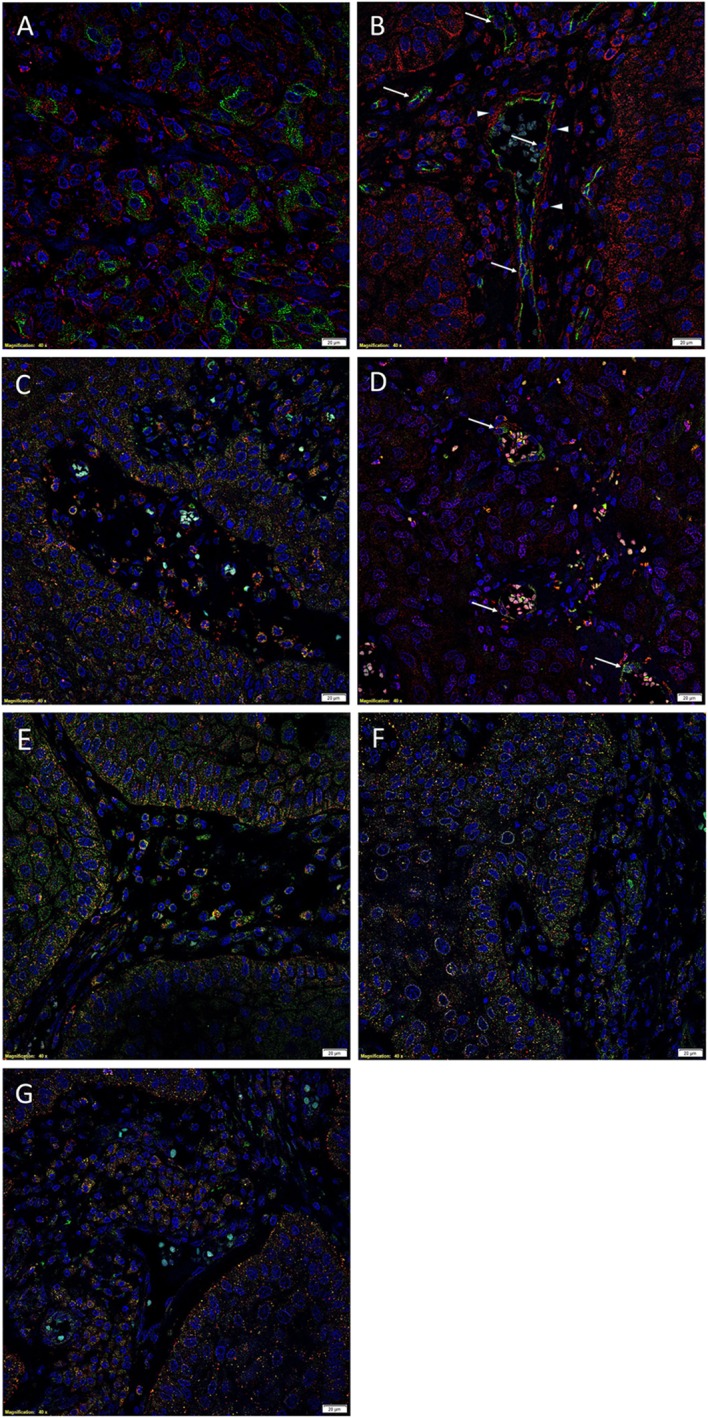
**Representative immunofluorescent immunohistochemical-stained sections of moderately differentiated buccal mucosal squamous cell carcinoma demonstrating PRR [(A), red] was expressed by cells within the tumor nests that stained positively for EMA [(A), green]**. PRR [**(B)**, red] was also expressed by the CD34^+^ [**(B)**, green] endothelium of the microvessels. In addition, RRR [**(C)**, red] was expressed by cells within the peri-tumoral stroma, which also expressed OCT4 [**(C)**, green]. ACE [**(D)**, green] was expressed on the endothelium of the microvessels, which also expressed SOX2 [**(D)**, red], within the peri-tumoral stroma. Cytoplasmic expression of ATIIR1 [**(E)**, green] and PRR [**(E)**, red] was demonstrated in cells within the tumor nests, the peri-tumoral stroma, and the endothelium of the microvessels within the peri-tumoral stroma. ATIIR2 [**(F,G)**, red] was expressed by cells within the tumor nests that expressed SALL4 [**(F)**, green] and OCT4 [**(G)**, green]. Cell nuclei were counterstained with 4′,6′-diamidino-2-phenylindole [**(A–G)**, blue]. Scale bars: 20 μm.

We have recently demonstrated the presence, within MDBMSCC, of an EMA^+^/SOX2^+^/SALL4^+^/OCT4^+^/pSTAT3^+^/NANOG^+^ CSC subpopulation within the tumor nests; an EMA^−^/SOX2^+^/OCT4^+^/pSTAT3^+^/NANOG^+^ CSC subpopulation within the peri-tumor stroma; and an EMA^−^/CD34^+^/SOX2^+^/OCT4^+^/pSTAT3^+^/NANOG^+^ CSC subpopulation localized to the endothelium of the microvessels within the peri-tumoral stroma (16). To investigate if the components of the RAS were expressed by one or more of these CSC subpopulations, we performed double IF IHC staining to investigate the expression patterns of PRR, ACE, ATIIR1, and ATIIR2.

(Pro)renin receptor (Figures 2A–C, red) was expressed by the EMA^+^ (Figure 2A, green) cells within the tumor nests, as well as the CD34^+^ endothelium (Figure 2B, green, *arrows*) and the outer pericyte layer (Figure 2B, red, *arrowheads*) of the microvessels and OCT4^+^ (Figure 2C, green) cells within the peri-tumoral stroma (16). ACE (Figure 2D, green, *arrows*) was expressed on the endothelium of the microvessels within the peri-tumoral stroma, which expressed SOX2 (Figure 2D, red), distinct from the tumor nests. Cytoplasmic expression of ATIIR1 (Figure 2E, green) was demonstrated on cells within the tumor nests and the endothelium of the microvessels within the peri-tumoral stroma expressing PRR (Figure 2E, red). ATIIR2 (Figures 2F,G, red) was expressed by cells within the tumor nests that have been shown to express SALL4 (Figure 2F, green) (16). Interestingly, cells within the peri-tumoral stroma that express OCT4 (16) also expressed ATIIR2 (Figure 2G, green). Separated IF IHC-stained images of Figure 2 are presented in Image S2 in Supplementary Material.

### Western Blotting

**Figure 3:**
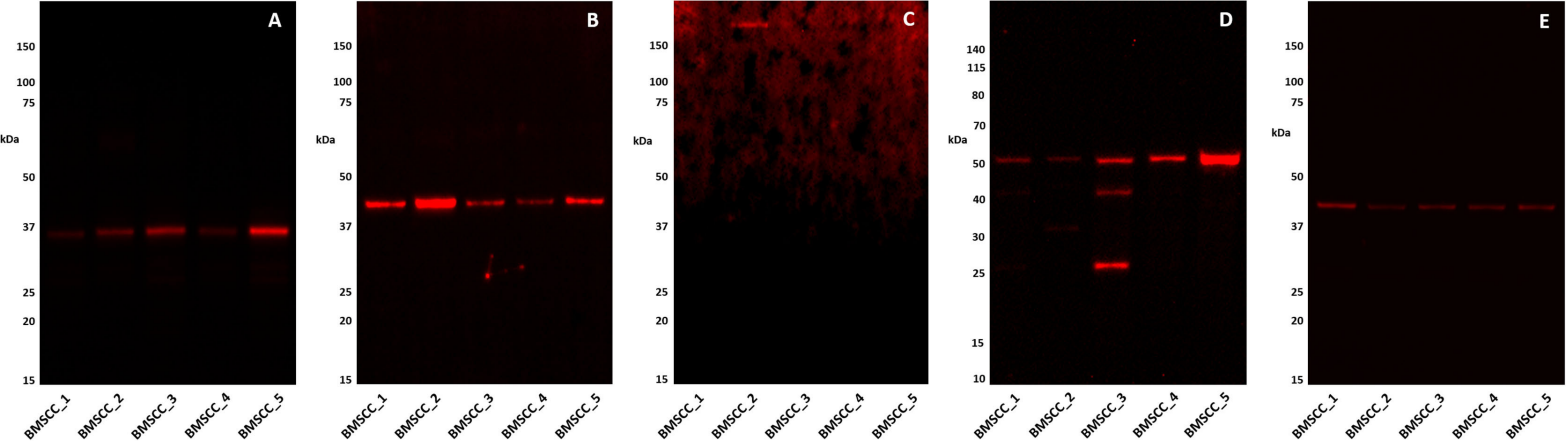
**Western Blot images of 1DE separated moderately differentiated buccal mucosal squamous cell carcinoma total protein extracts probed for PRR (A), ATIIR1 (B), ACE (C), ATIIR2 (D), and detected with HRP conjugated goat anti-rabbit (A,B,D) or donkey anti-goat (C), or rabbit anti-rabbit (E) secondary antibody**. β-actin **(E)** was used as the loading control and detected using Alexafluor^®^ 647 rabbit anti-mouse secondary antibody.

Western Blot analysis confirmed the presence of PRR (Figure 3A) and ATIIR1 (Figure 3B) within the extracts of all five MDBMSCC samples from the original cohort of patients used for DAB IHC staining, at their expected molecular weight of 39 and 43 kDa, respectively. ACE (Figure 3C) was detected in only one of the five MDMBSCC samples analyzed, at the expected molecular weight of 195 kDa. High image exposure was required to clearly visualize the ACE signal in the MDMBSCC sample, which implies that ACE was present at very low abundance. ATIIR2 was detected in all five MDBMSCC samples at approximately 51 kDa (Figure 3D), which is larger than the native form of the protein and may signify detection of a glycosylated isoform. A band at approximately 41 kDa was detected in one of the five total protein extracts (Figure 3D, BMSCC_3) that may correspond to the native protein (27). Detection of β-actin confirmed approximately equal total protein loading across all five samples (Figure 3E).

### NanoString Gene Analysis

**Figure 4:**
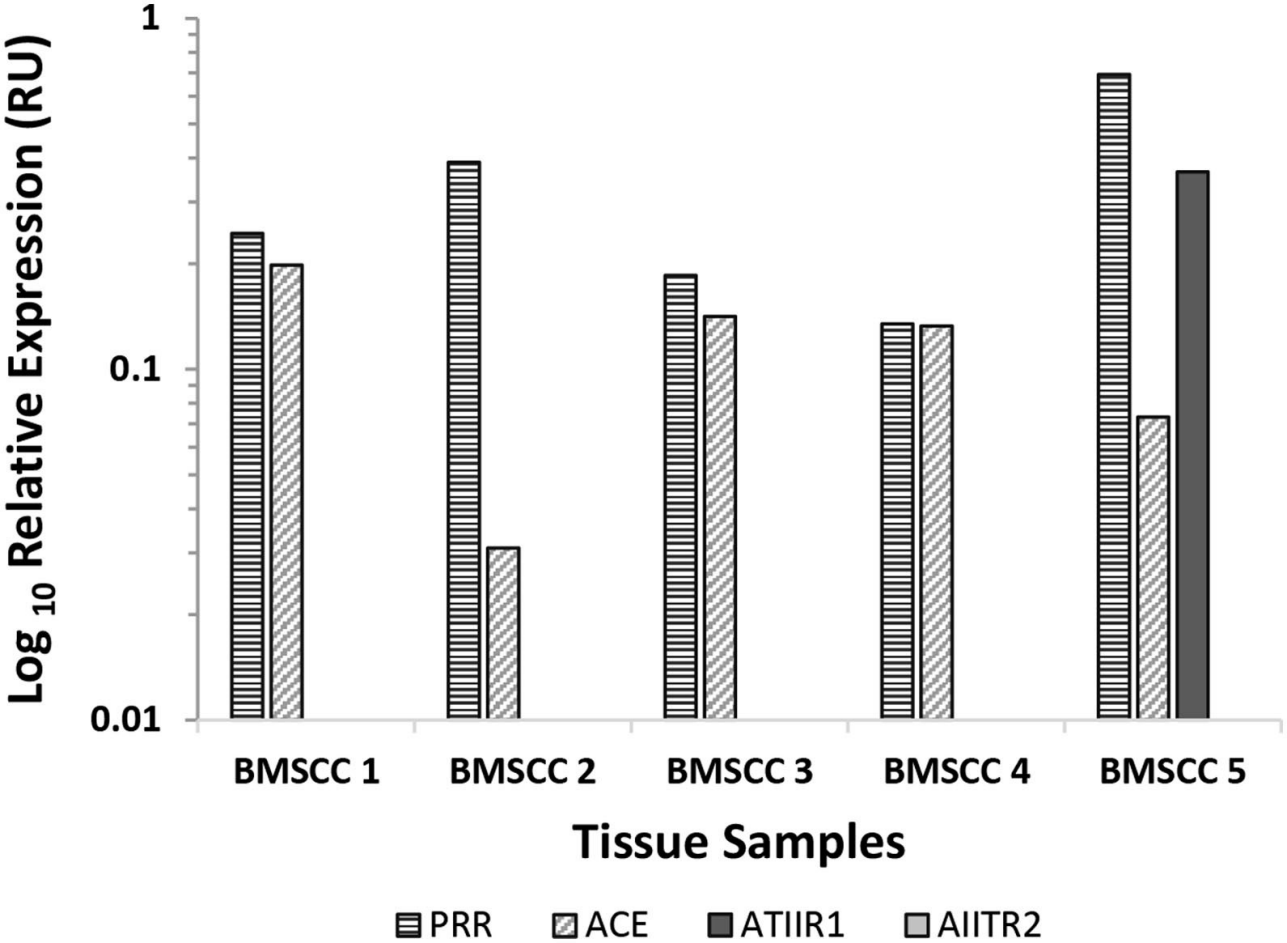
**Relative expression of mRNA transcripts of the components of the renin–angiotensin system in five moderately differentiated buccal mucosal squamous cell carcinoma samples**. Expression is depicted in relative units (RU) as a ratio to the GUSB housekeeper. Transcriptional profiling confirmed the presence of PRR and ACE mRNA in all five samples and ATIIR1 in one sample, but ATIIR2 mRNA was below the detectable level within all five samples.

NanoString gene analysis of the components of the RAS: PRR, ACE, ATIIR1, and ATIIR2, was performed in five samples of MDBMSCC from the original cohort of six patients used for DAB IHC staining, normalized against the housekeeping gene, GUSB. Transcriptional profiling confirmed the presence of PRR and ACE mRNA in all five samples and ATIIR1 in one sample, but ATIIR2 mRNA was below the detectable level within all five samples (Figure 4).

## Discussion

The novel finding of the expression pattern of PRR, ATIIR1, ATIIR2, and ACE on CSC subpopulations within MDBMSCC provides insights into the biology of this aggressive tumor. It is noteworthy that the CSC subpopulation within the peri-tumoral stroma does not express EMA. This CSC subpopulation may represent a “normal” stem cell population or cells that have undergone an epithelial to mesenchymal transition (EMT) (28), with the endothelial population possibly reflecting the phenomenon of vascular mimicry (29).

It is intriguing that although IHC staining demonstrated the expression of both ATIIR1 and ATIIR2, supported by WB detection of bands at the expected size, mRNA expression for ATIIR2 was not detected. This may be due to rapid degradation of mRNA of this protein.

Recent literature suggests that the RAS may play a role in cancer growth and metastasis, especially in promoting cell proliferation and angiogenesis (25, 30), with a review highlighting a role for the RAS in other cancers, predominantly in cellular proliferation (28). We have demonstrated an interesting expression pattern, within MDBMSCC; the components of the RAS on the CSC subpopulations within the tumor nests, peri-tumoral stroma, and the endothelium of the microvessels within the peri-tumoral stroma.

The expression of the ESC marker SOX2 on the endothelium adjacent to the tumor nests is consistent with our recent publication demonstrating expression of the stem cell markers SALL4 and OCT4 (16). These findings and the observation of the expression of PRR and ACE on the endothelium is intriguing and may reflect a primitive phenotypic endothelium similar to that reported in colorectal cancer (29), although this remains a topic for future investigation.

While the analysis of normal buccal mucosa would strengthen the interpretation of our data, these novel findings of the localization of the components of the RAS to the CSC subpopulations within MDBMSCC mirror our similar finding within OTSCC (17, 31). Although we have not demonstrated the functionality of these receptors in this report, it is exciting to speculate CSCs as a potential novel therapeutic target by modulation of the RAS using existing anti-hypertensive drugs, such as aliskiren, which targets renin; β-blockers, which block the production of (pro)renin and so decrease levels of renin; ACE inhibitors and ATII receptor blockers (32–35).

The relatively small sample numbers and the lack of functional data for these receptors in this report are limitations of the study and demonstrate the need for further work to better understand the precise regulatory function of the RAS on the CSC subpopulations, which may lead to an effective treatment for BMSCC and other cancers.

## Ethics Approval

Central Regional Health and Disability Ethics Committee (ref. no. 12/CEN/74).

## Take Home Messages

1. The components of the RAS: PRR, ACE, ATIIR1, and ATIIR2 are expressed by MDBMSCC.
2. PRR is localized to the CSC subpopulations within the tumor nests, the peri-tumoral stroma, and the endothelium of the microvessels within the peri-tumoral stroma, between the tumor nests.
3. ATIIR1 and ATIIR2 are localized to the CSC subpopulations within the tumor nests and the peri-tumoral stroma.
4. ACE is localized to the endothelium of the microvessels within the peri-tumoral stroma.
5. These novel findings suggest CSCs within this tumor as a potential therapeutic target by modulating the RAS.

## Author Contributions

TI and STT proposed the study hypothesis and designed the study. TF, HHY, HDB, STT, and TI interpreted the IHC data. TF, HHY, and JCD performed WB and interpreted the data. AMC processed the tissues for NanoString analysis and interpreted the data. TF, TI, PFD, and STT drafted the manuscript. All authors approved the manuscript.

## Conflict of Interest Statement

The authors declare that the research was conducted in the absence of any commercial or financial relationships that could be construed as a potential conflict of interest. TI, PD, and ST are inventors of the PCT patent application (No. PCT/NZ2015/050108) Cancer Diagnosis and Therapy.

## Supplementary Material

Image S1 **Representative DAB IHC-stained sections of positive control human tissues showing positive staining for PRR in human placenta (A); and ACE (B), ATIIR1 (C), and ATIIR2 (D) in liver**. A MDBMSCC sample was used as negative control by the omission of the primary antibody **(E)**. Cell nuclei were counterstained with hematoxylin [**(A–E)**, blue]. Original magnification: 400×.

Image S2 **Split IF IHC images of Figure 2 demonstrating expression of PRR [(A), red] and EMA [(B), green]; PRR [(C), red] and CD34 [(D), green]; PRR [(E), red] and OCT4 [(F), green]; SOX2 [(G), red] and ACE [(H), green]; PRR [(I) red] and ATIIR1 [(J), green]; SALL4 [(K), green] and AIITR2 [(L), red]; OCT4 [(M), green] and AIITR2 [(L), red]**. Cell nuclei were counterstained with 4′,6′-diamidino-2-phenylindole [**(A–N)**, blue]. Scale bars: 20 μm.
