# Supplementary figures and images for: Cancer Stem Cells in Moderately Differentiated Buccal Mucosal Squamous Cell Carcinoma Express Components of the Renin–Angiotensin System
Source: Front Surg. 2016 Sep 27;3:52. doi: 10.3389/fsurg.2016.00052 (PMC5037224; doi:10.3389/fsurg.2016.00052)

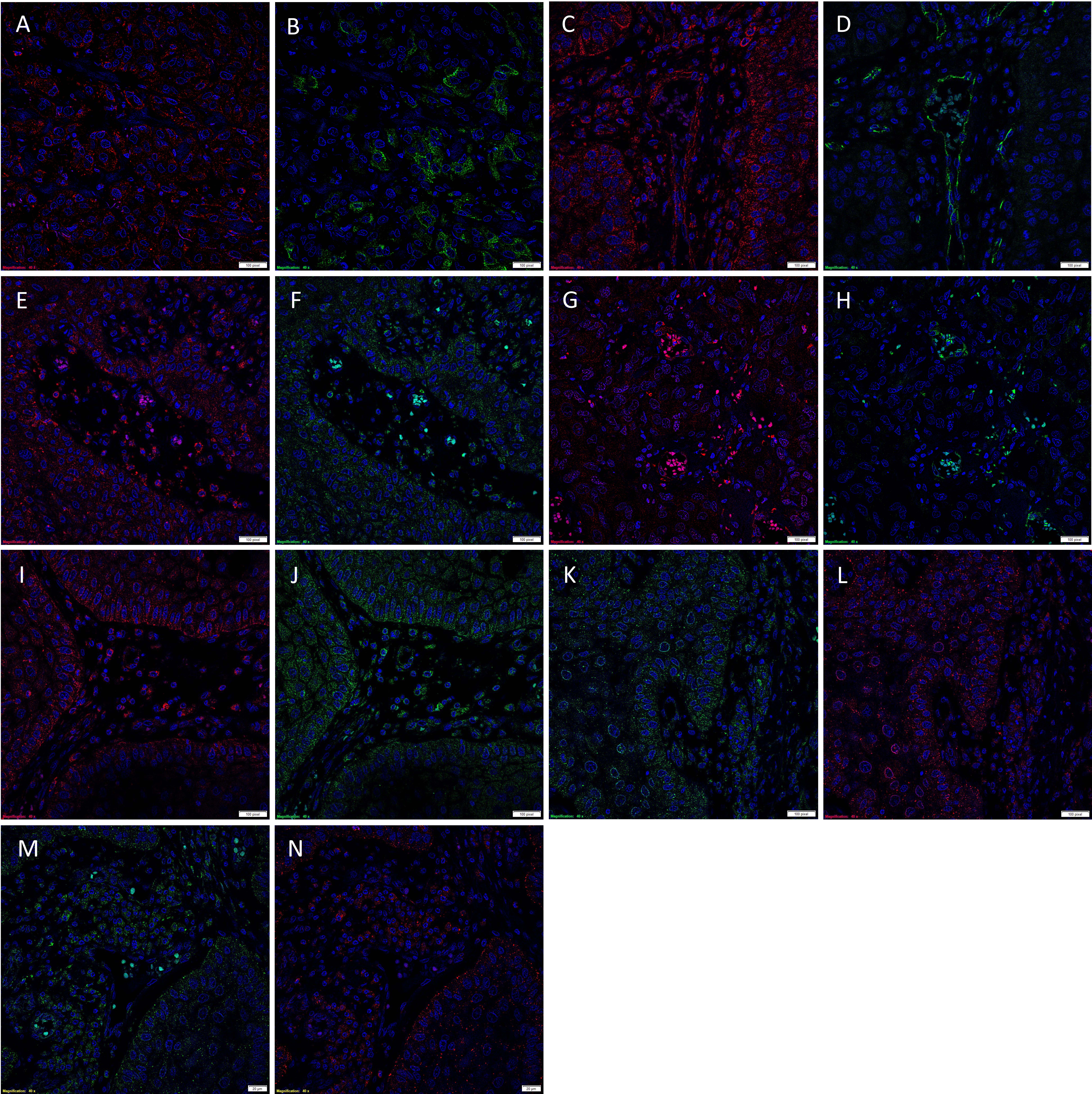

Supplement: Image S2 — Split IF IHC images of Figure 2 demonstrating expression of PRR [(A), red] and EMA [(B), green]; PRR [(C), red] and CD34 [(D), green]; PRR [(E), red] and OCT4 [(F), green]; SOX2 [(G), red] and ACE [(H), green]; PRR [(I) red] and ATIIR1 [(J), green]; SALL4 [(K), green] and AIITR2 [(L), red]; OCT4 [(M), green] and AIITR2 [(L), red]. Cell nuclei were counterstained with 4′,6′-diamidino-2-phenylindole [(A–N), blue]. Scale bars: 20 μm. [file Image_2.jpg]
